# Frequency-specific electrophysiologic correlates of resting state fMRI networks

**DOI:** 10.1016/j.neuroimage.2017.01.054

**Published:** 2017-01-31

**Authors:** Carl D. Hacker, Abraham Z. Snyder, Mrinal Pahwa, Maurizio Corbetta, Eric C. Leuthardt

**Affiliations:** a Department of Neurosurgery, Washington University School of Medicine, Campus Box 8225, United States; b Department of Radiology, Washington University School of Medicine, Campus Box 8225, United States; c Department of Neurology, Washington University School of Medicine, Campus Box 8225, United States

## Abstract

Resting state functional MRI (R-fMRI) studies have shown that slow (< 0.1 Hz), intrinsic fluctuations of the blood oxygen level dependent (BOLD) signal are temporally correlated within hierarchically organized functional systems known as resting state networks (RSNs) (Doucet et al., 2011). Most broadly, this hierarchy exhibits a dichotomy between two opposed systems (Fox et al., 2005). One system engages with the environment and includes the visual, auditory, and sensorimotor (SMN) networks as well as the dorsal attention network (DAN), which controls spatial attention. The other system includes the default mode network (DMN) and the fronto-parietal control system (FPC), RSNs that instantiate episodic memory and executive control, respectively. Here, we test the hypothesis, based on the spectral specificity of electrophysiologic responses to perceptual vs. memory tasks (Klimesch, 1999; Pfurtscheller and Lopes da Silva, 1999), that these two large-scale neural systems also manifest frequency specificity in the resting state. We measured the spatial correspondence between electrocorticographic (ECoG) band-limited power (BLP) and R-fMRI correlation patterns in awake, resting, human subjects. Our results show that, while gamma BLP correspondence was common throughout the brain, theta (4–8 Hz) BLP correspondence was stronger in the DMN and FPC, whereas alpha (8–12 Hz) correspondence was stronger in the SMN and DAN. Thus, the human brain, at rest, exhibits frequency specific electrophysiology, respecting both the spectral structure of task responses and the hierarchical organization of RSNs.

## Introduction

Intrinsic brain activity has emerged as a major focus of systems neuroscience research (Raichle, 2009). Resting state, i.e., task-free, functional magnetic resonance imaging (R-fMRI) currently is the primary technique used in the investigation of intrinsic brain activity (Smith et al., 2013). On the basis of R-fMRI studies, it is now established that slow (< 0.1 Hz), intrinsic fluctuations of the blood oxygen level dependent (BOLD) signal are temporally correlated within spatially distributed functional systems. This phenomenon is widely known as functional connectivity. The associated topographies are known as resting state networks (RSNs) or, equivalently, intrinsic connectivity networks (ICNs) (Zielinski et al., 2010).

R-fMRI RSNs are hierarchically organized (Cordes et al., 2002; Gleiser and Spoormaker, 2010). The RSN hierarchy splits at the highest level into two “systems,” originally referred to as “task positive” vs. “task negative” (Fox et al., 2005) but more recently conceptualized as the “extrinsic” vs. “intrinsic” systems (Doucet et al., 2011). The intrinsic system includes the default mode network (DMN), which is recruited by episodic memory (Buckner et al., 2008) and social cognition (Adolphs, 2009), and the fronto-parietal control network (FPC), which is recruited by executive control tasks (Dosenbach et al., 2006; Fedorenko et al., 2013). The extrinsic system includes visual areas, the somatomotor network (SMN), which is recruited during movement execution and planning, and the dorsal attention network (DAN), which mediates top-down control of spatial attention (Corbetta and Shulman, 2002). This distinction between the intrinsic vs. extrinsic functional systems is supported by meta-analyses of task activation studies (Shulman et al., 1997a; Shulman et al., 1997b) as well as hierarchical analyses of R-fMRI (Doucet et al., 2011; Lee et al., 2012).

Although it is generally assumed that there exist electrophysiological correlates of RSNs, the fundamental nature of these correlates remains under investigation (Scholvinck et al., 2013). Electrocorticographic (ECoG) recordings from the surface of the brain provide a means of studying the electrophysiologic correlates of RSNs at a temporal resolution inaccessible to fMRI. Task-based studies in primates (Goense and Logothetis, 2008) as well as humans (Koch et al., 2009; Miller et al., 2014) suggest that the most robust electrophysiological correlate of task engagement is increased wide band power, nominally in the range of 40–150 Hz. Not surprisingly, induced broadband ECoG activity also robustly corresponds to task evoked BOLD fMRI responses (Winawer et al., 2013). Indeed, mapping of task-induced, invasively recorded gamma band (nominally, 60–130 Hz) activity is increasingly used as a means of localizing function in neurosurgical patients (Qian et al., 2013).

Comparatively less is known about the spectral characteristics of resting state electrophysiological activity in relation to R-fMRI. For technical as well as patient safety reasons, studying resting-state electrophysiology in relation to R-fMRI generally involves acquiring each modality separately and then evaluating the spatial correspondence between the electrophysiological and BOLD R-fMRI temporal correlation structures.^2^ The correlation structure of spontaneous ECoG activity has been computed in two ways: (1) directly from local field potentials or (2) as the temporal correlation of slow modulations in the band limited power (BLP) envelopes of fast LFP oscillations (Engel et al., 2013; He et al., 2008; Leopold et al., 2003). Here, we exclusively use the second, BLP-based method. Prior human studies suggest that the spatial correspondence between ECoG BLP temporal correlations and BOLD R-fMRI temporal correlations is best observed with BLP evaluated in the gamma range (nominally, 70–110 Hz) (He et al., 2008; Keller et al., 2013). However, other observations suggest that BLP correlations may be, at least under some circumstances, more robust at slower carrier frequencies, i.e., in the theta and alpha ranges (4–13 Hz) (Wang et al., 2012). Thus, which carrier frequencies exhibit BLP envelope correlations that most closely correspond to the topography of R-fMRI RSNs remains uncertain. Here, we report a systematic investigation of this question in human epilepsy patients.

Clues regarding what might be expected derive from a large body of work that has associated alpha (8–12 Hz) desynchronization (power decreases) with motor (Pfurtscheller and Lopes da Silva, 1999) and perceptual/attentional tasks (Klimesch, 1999; Sauseng et al., 2009). In contrast, executive control (Sederberg et al., 2003), working memory (Bastiaansen et al., 2008; Raghavachari et al., 2001), and episodic memory tasks (Fell et al., 2011), induce theta (4–8 Hz) synchronization, although not necessarily theta BLP increases. It is generally agreed that engagement of the extrinsic system predominantly *suppresses* alpha oscillations (Klimesch, 1999; Miller et al., 2007; Sederberg et al., 2003). Engagement of the intrinsic system has been variably reported as *enhancing* (Colgin, 2013) or *suppressing* (Burke et al., 2015; Crespo-García et al., 2016; Greenberg et al., 2015) theta oscillations. However, if these modulations (of either sign) are spontaneously coordinated across regions (i.e., in the task-free state), we may expect to observe spectrally specific spatial correspondences between BLP correlations and BOLD fMRI RSNs. Moreover, this spectral specificity should depend on large-scale functional system, i.e., the intrinsic vs. the extrinsic system.

## Methods

### Participants

All participants were patients at Barnes Jewish Hospital or St. Louis Children’s Hospital with drug-resistant epilepsy undergoing electrocorticographic (ECoG) monitoring to localize seizure foci. All participants provided informed consent with oversight by the local Institutional Review Board in accordance with the National Institutes of Health guidelines and the ethical standards of the Declaration of Helsinki. Participants were selected from a database of 25 patients who had undergone invasive monitoring and provided consent for ECoG and fMRI research. Initial inclusion criteria were at least 4 days of clinical ECoG recordings as well as pre-operative structural and functional MRI and post-implant X-ray computed tomography (CT) images were acquired. Subjects were excluded based on 4 categories of criteria. 1) Available data: no post-operative CT (n=3), no resting fMRI (n=3, 2 of which were contra-indicated due to presence of vagal nerve stimulators), clinical ECoG recordings not available (n=2). 2) Pathology: extensive cortical dysplasia (n=1) or neoplasia (n=1). 3) Post-operative complications: hematoma (n=1). 4) Cognition: IQ < 75 (n=2), congenital blindness (n=1), aphasia (n=1). 5) Data quality: fMRI head motion (< 75% usable frames, n=3; see Image Preprocessing), insufficient number of seizure free epochs (n=1). Six subjects passed all inclusion criteria (see Table S1 for subject profiles).

### fMRI acquisition and preprocessing

Structural and functional imaging was performed with a 3T Tim Trio Scanner (Siemens, Erlangen, Germany) using product sequences. Functional images were acquired using a BOLD contrast sensitive gradient echo echo-planar sequence (90° flip angle, (4 mm)^3^ voxel size, 2.16 s TR; see also Table S2) during which participants were instructed to fixate on a visual cross-hair, remain still and not fall asleep. Anatomical imaging included one sagittal T1-weighted magnetization prepared rapid gradient echo (MP-RAGE) scan and one T2-weighted scan.

fMRI preprocessing proceeded as previously described (Hacker et al., 2013) with the addition of image distortion correction using the FUGUE module in FSL (Jenkinson et al., 2012). Field maps were approximated using the technique described by Gholipour et al. (2008). Distortion correction and motion correction were combined in one resampling step to generate volumetric time-series in Talairach atlas space (3×3×3 mm cubic voxels). Additional preprocessing in preparation for FC analyses included motion censoring based on the DVARS (temporal derivative of RMS BOLD signal across voxels) measure (Power et al., 2012; Smyser et al., 2010). Motion censoring was computed before de-noising to exclude frames (volumes) with “cosmetically” improved DVARS values but retained artifact (Power et al., 2014). The DVARS censoring threshold was set at 0.5% root-mean-square frame-to-frame BOLD signal change (Power et al., 2014) following 6 mm spatial pre-blur in each direction (used only for frame censoring). Epochs containing fewer than 10 contiguous frames meeting the DVARS criterion were excluded from the functional connectivity computations. The fraction of censored data from each participant is listed in Table S2.

Following motion censoring, the retained frames were made zero-mean within each voxel but the data were not otherwise temporally or spatially filtered. Initial de-noising was accomplished using a strategy similar to CompCor (Behzadi et al., 2007). Nuisance regressors were derived from white matter and ventricle masks, segmented in each individual using FreeSurfer (Fischl, 2012), then spatially resampled in register with the functional data. Nuisance regressors also were extracted from voxels in the extra-axial CSF space exhibiting high (>2.5%) temporal standard deviation. Nuisance regressors also were derived from rigid body head motion correction. Following nuisance regression, the volumetric timeseries were further de-noised using an ICA regression approach. Because of the small number of subjects (n=6) in this study, components for all subjects were manually classified according to established criteria (Kelly et al., 2010). Unambiguously artifactual components were eliminated by linear regression (Fig. S1). The global signal (GS) averaged over the whole brain and its temporal derivative were removed by linear regression (see Keller et al. (2013)), for discussion of GS regression in ECoG:fMRI comparisons). fMRI timeseries were prepared for comparison to ECoG data by ribbon-constrained volume to surface resampling using the Human Connectome Project pipeline (Glasser et al., 2013).

Resting state network topographies were computed in each subject using a supervised classification method (Hacker et al., 2013). Briefly, this technique employs a neural network (specifically, a multi-layer perceptron performing non-linear regression) that has been trained to associate correlation maps of a standard set of task-derived seed regions with pre-defined RSN labels. We classified seed-based fMRI correlation maps generated at every brain locus to produce RSN topographies throughout the brain.

### Anatomical registration of electrodes

After electrode implantation (Fig. S2A), a post-operative CT image (Fig. S2B) was registered (6-parameter rigid body) to the pre-operative T1-weighted image (Fig. S2C). Post-implantation, ECoG electrodes generally are displaced downwards relative to the pre-operative brain surface owing to traction generated by dural over-sewing. To correct this displacement, electrodes were projected to a highly smoothed pial surface (a modified FreeSurfer segmentation) using normal vectors computed from grid and strip geometry (Fig. S2D). Following this correction, total electrode localization error was estimated as ~2 mm. This precision is comparable to previous results (Hermes et al., 2010).

### Imaging-based electrode exclusion

fMRI data characteristically are compromised by focal areas of signal dropout caused by magnetization susceptibility inhomogeneities (Ojemann et al., 1997). To accommodate this problem, an intensity iso-surface was computed at 50% of the mean brain value. Electrodes more than 5 mm distant from the iso-surface were excluded from the ECoG:fMRI analyses (see Table S1, Rejected (Imaging) column).

### Sampling BOLD data to electrodes

Our work incorporates several methodological innovations in the comparison of ECoG and fMRI data designed to enhance specificity. To improve registration of fMRI data with electrodes, we incorporated knowledge of the local cortical surface geometry in weighting the contribution of fMRI signal at each electrode. fMRI noise was also reduced by geodesic smoothing on the cortical surface (Glasser et al., 2013). These maneuvers, in combination, reduced cross-gyral contamination and improved spatial specificity while preserving the fMRI signal to noise ratio. After registration of electrode coordinates to individual cortical surfaces (Fig. S2E), surface fMRI timeseries were projected to each electrode according to the expected relative contribution of each brain locus modeled under electrostatic assumptions. Specifically, the cerebral grey matter was modeled as a sheet of diploes oriented normal to the cortical mid-thickness surface. The angular component of the dipole was ignored because it was empirically determined that small errors in electrode registration relative to the cortical ribbon geometry led to numerical instability. Thus, the forward solution was modeled by the inverse square of the distance from cortical mid-thickness surface vertices (*r_j_*) to points on the electrode surface (*r_i_*). The contribution to electrode *e* of cortical surface vertices *j*were found by integrating over all elements *i* of the electrode surface *S_e_*:

(1) $$w_{ej}={\int_{i\in S_{e}}\left\|r_{i}-r_{j}\right\|}^{-2}dS_{e}$$

The surface map of weights for electrode *e*, *w_e_*_•,_ was normalized to unit sum:

(2) $${\bar{w}}_{ej}=\frac{w_{ej}}{\sum_{j\in s}w_{ej}}$$

**Fig. 1:**
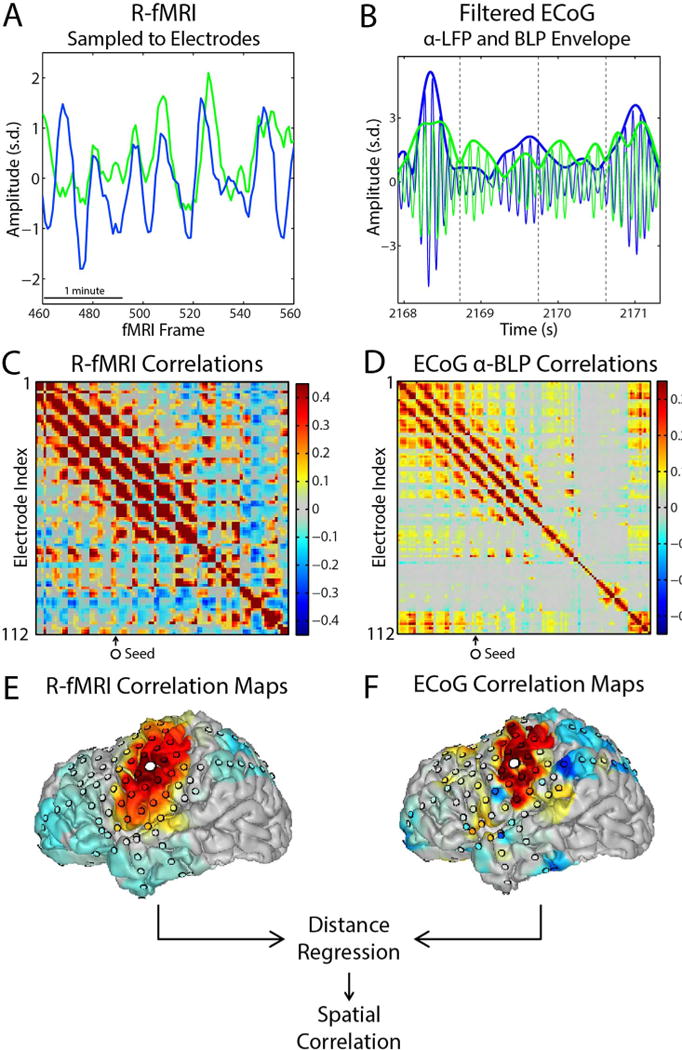
Methodology for spatially comparing ECoG and fMRI correlation maps. A. fMRI timeseries are sampled to electrodes according to the relative sensitivity to dipoles across the cortical surface (see Methods and Fig. S2 and S3). B. ECoG data are band-pass filtered and rectified to compute the band-limited power (BLP) envelope. The BLP is further bandpass filtered to isolate particular envelope frequency components (see Methods). C and D. Temporal correlations are computed between all pairs of electrodes for both fMRI and ECoG data. Correlation matrices are shown after the removal of the systematic relationship between correlation and inter-electrode distance in both modalities (see Fig. S3 and S4 for methodology for producing these correlation matrices). E and F. Each column of the correlation matrix corresponds to one seed-based correlation map (illustrated on brain surfaces for ECoG and fMRI data, large marker: seed electrode). Local correlations are removed (‘distance regression,’ see Fig. S5). The (spatial) correlation is computed between column-vectors corresponding to the same seed to obtain a single measure of ECoG:fMRI correspondence at a particular seed electrode and ECoG frequency.

These weights were expressed as a matrix, *W*, of dimension [electrodes × cortical vertices]. *W* was used to sample cortical surface maps of each frame of the fMRI timeseries, *f* (*t*), onto the space of electrodes: *f_e_* (*t*) = *Wf* (*t*). *f_e_* (*t*) was subsequently used to compute the fMRI temporal correlation matrix in electrode space, thereby ensuring that the fMRI and ECoG data were analyzed identically. The same sampling weights were used for sampling RSN network membership estimates to electrodes for classifying electrodes into RSNs. The weight matrix *W* was also used for illustrative purposes to create surface displays of correlation maps computed in electrode space, e.g., the topographies in Fig. 1.

### ECoG data acquisition and preprocessing

Implanted electrodes (platinum, 4 mm, 2.3 mm exposed, PMT corporation) were 8×8 or 6×8 grids (with 1 cm spacing) and strips (1×4, 1×6, or 1×8), placed subdurally facing the cortical surface. A separate strip facing the skull served as ground and reference for the amplifier (Proamp, Lamont Medical Inc). Data were recorded at 512 Hz (except PT1, where the sampling rate was 200 Hz) with a 0.1–500 Hz band-pass filter (18-dB/octave roll-off). Data were screened for channels with excessive noise and epochs with excessive environmental noise across all channels; channels exhibiting inter-ictal activity also were excluded.

Long intervals of wakefulness with minimal motor activity (eating, talking, arm or head movements) were included. Sleep epochs were defined behaviorally with the video records; additionally, periods of sustained delta power (> 20% power in the 0.5–4 Hz range) were identified as slow wave sleep (SWS) and excluded. The present ECoG:fMRI analyses include only ECoG recordings at least 30 min separated from behaviorally identified sleep (from video recordings) or electrophysiologically identified SWS. Ictal events were identified by clinical staff. ECoG data recorded up to 2 h following ictal events were excluded.

ECoG signals were referenced to a common average. A de-spiking function, *f* (*x*)=*a*•*tan*^−1^(*x*/*a*), where *a* is 5 s.d. of the signal, was applied to attenuate transient artifacts from medical devices (e.g., IV pumps). Data were further inspected for artifact in the time-frequency domain. ECoG signals were decomposed into frequency components by zero-phase digital filtering using a 2nd order Butterworth filter in the forward and reverse directions (effectively, 4th order). Frequency bins were logarithmically spaced with bin edges defined as 2k Hz, where k ranges from 0 to 7 in increments of 0.1. The filtered signals were squared to produce instantaneous power. In other applications, a low-pass filter could be applied to recover the full signal envelope (equivalent to envelopes obtained using the Hilbert transform). However, we wanted to subdivide envelope modulations into distinct frequency bands. Accordingly, we applied a second band-pass filter to the squared signals obtained in the previous step to obtain ‘envelope frequency’ bins. Envelope frequency bins were logarithmically spaced with bin edges defined as 10^k^ Hz, where k ranges from −2.5 to 1.25 in increments of 0.25. Measurable envelope frequencies cannot exceed the bin width applied to the carrier frequency. Thus, for example, for BLP bin spanning 104–111 Hz, the envelope signal was filtered to isolate frequency components ranging from 0.003 Hz to a maximum of 7 Hz (i.e., the width of the 104–111 Hz carrier frequency bin).

### Computation of ECoG:fMRI correspondence spectra

The objective of the present work is to investigate how the spatial correspondence between ECoG BLP correlations and BOLD RSNs depends on electrophysiological spectral content. To this end, we compare the large-scale topography of seed-based correlations of fMRI time series and ECoG signals as a function of ECoG BLP carrier and envelope frequencies. We define the results of this spatial comparison as an ECoG:fMRI correspondence spectrum, computed for each seed locus.

Pearson product-moment correlation maps for each type of data were computed across all pairs of electrodes, treating the fMRI and ECoG BLP timeseries identically. Before computing ECoG:fMRI correspondence spectra, we took into account that both ECoG and BOLD correlations are systematically greater at short distances. Thus, dependence on distance of both ECoG and BOLD fMRI correlations was removed by B-spline regression prior to computing ECoG:fMRI correspondence spectra. This procedure removes local correlations within each modality (fMRI and ECoG; see Figs. S3 and S4), which, if present, would produce non-specific cross modal correspondence at all electrodes and at all frequencies. This distance regression strategy removes radially symmetric correlations and is spatially stationary (similar in principle to a spatial high-pass filter). Hence, it is inherently unbiased with respect to specific RSNs (see on-line Supplementary Discussion and Fig. S5 for addition details). Empirically, distance regression removes a baseline level of ECoG:fMRI correspondence that is present at all frequencies thereby revealing increased spectral focality (Fig. S5.C vs. S5.F). Finally, ECoG:fMRI correspondence was computed as the Fisher z-transformed spatial correlation of the ECoG band-limited power (BLP) and fMRI correlation maps. Correspondence was computed parametric in carrier and envelope frequency, thereby producing carrier frequency×envelope frequency correspondence spectra for each electrode (e.g., Fig. 1C). As a final step, correspondence spectra were smoothed with a moving average filter (span of 3 bins) in log frequency space.

## Results

To enable direct comparison of resting state ECoG vs. BOLD fMRI temporal correlations, preprocessed fMRI timeseries (see Methods) were projected onto the brain surface and subsequently resampled at electrode loci (see Figs. S1–S3). Surface-to-electrode BOLD signal resampling was computed according to the expected electrophysiologic contribution of each surface locus modeled as a transcortical dipole. ECoG signals were referenced to the common mean (excluding noisy and ictal electrodes) and band-pass filtered at logarithmic intervals to isolate particular carrier frequencies; these band-limited signals were squared and then filtered to isolate specific modulation frequencies of the BLP signal derived from a given carrier frequency band (see Methods for further details). Pearson product-moment temporal correlations were computed for all electrode pairs, treating the fMRI and ECoG BLP timeseries identically. ECoG:fMRI correspondence was computed as the spatial correlation of the ECoG BLP and fMRI (temporal) correlation maps. Fig. 1 illustrates this analysis scheme for one seed electrode at one frequency.

**Fig. 2:**
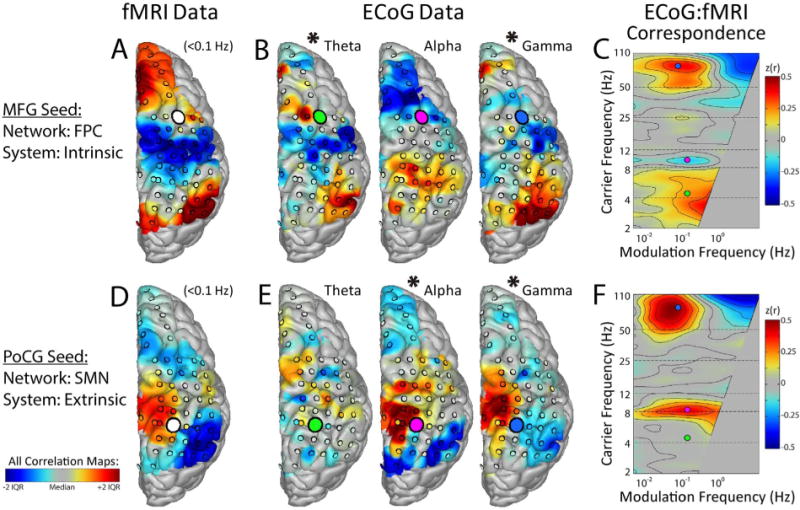
Spatial correspondence of ECoG and fMRI correlation maps in a single subject. A. Seed-based fMRI correlation map for a seed region (white circle) overlying the middle frontal gyrus (MFG), within the fronto-parietal control system (FPC). The correlation map rendering is limited to regions with electrode coverage. B. Seed-based ECoG BLP correlation maps for the same seed location as in A. C. ECoG:fMRI correspondence assessed by spatial correlation of (A) and (B), parametric in ECoG BLP carrier and modulation frequencies. Colored circles indicate frequencies for exemplars in (B). Note that ECoG correlation maps for theta and gamma frequencies (asterisks) for this seed region in (B) are similar to the fMRI correlation map in (A). Bottom half (D,E,F) illustrates results as per A,B,C for a seed region in the post-central gyrus (PoCG), within the sensorimotor network (SMN). Note that correlation maps for the SMN seed at alpha (rather than theta) and gamma frequencies (asterisks in E) are similar to the fMRI correlation map in (D). Thus, the frequency specific ECoG:fMRI similarity produces complimentary peaks of spatial correlation in the theta and alpha ranges in panels C and F, respectively.

Exemplar results are shown in Fig. 2. Panel A shows the fMRI correlation map obtained with a seed electrode overlying the middle frontal gyrus (MFG), a locus within the fronto-parietal control RSN, which is a component of the intrinsic system. Positive correlation was observed with signals in lateral parietal cortex (frontoparietal control network) and superior frontal cortex (default mode), and negative correlation with precentral/postcentral cortex (motor), and intraparietal sulcus (dorsal attention). Panel B shows the corresponding ECoG BLP seed-based correlation maps obtained with the same seed at various carrier frequencies. As is evident in panels A and B, the topography of theta and gamma (asterisks) but not alpha BLP correlations was spatially similar to the topography of BOLD fMRI temporal correlations. A quantitative summary of these findings is presented Fig. 2C, which shows the ECoG:fMRI correspondence spectrum (across carrier×modulation frequencies). ECoG:fMRI correspondence was modestly dependent on modulation frequency but strongly dependent on carrier frequency. Thus, BLP spectral specificity appears in the carrier×modulation display as broad horizontal bands with peaks within the high gamma (50–100 Hz) and theta (4–8 Hz) carrier frequencies. Panels D-F illustrate complementary results, obtained with an electrode seed overlying the post-central gyrus (PoCG), a locus within the SMN, which is a component of the extrinsic system. This locus showed strong ECoG:fMRI correspondence with alpha (8–12 Hz) and gamma but not theta frequencies. Thus, whereas both seeds exhibited similar resting state BOLD fMRI and BLP correlation topographies at high gamma frequencies, intrinsic vs. extrinsic spectral specificity was observed at theta and alpha BLP frequencies, respectively. See Fig. S6 for additional intrinsic system and extrinsic system exemplars for all other subjects.

**Fig. 3:**
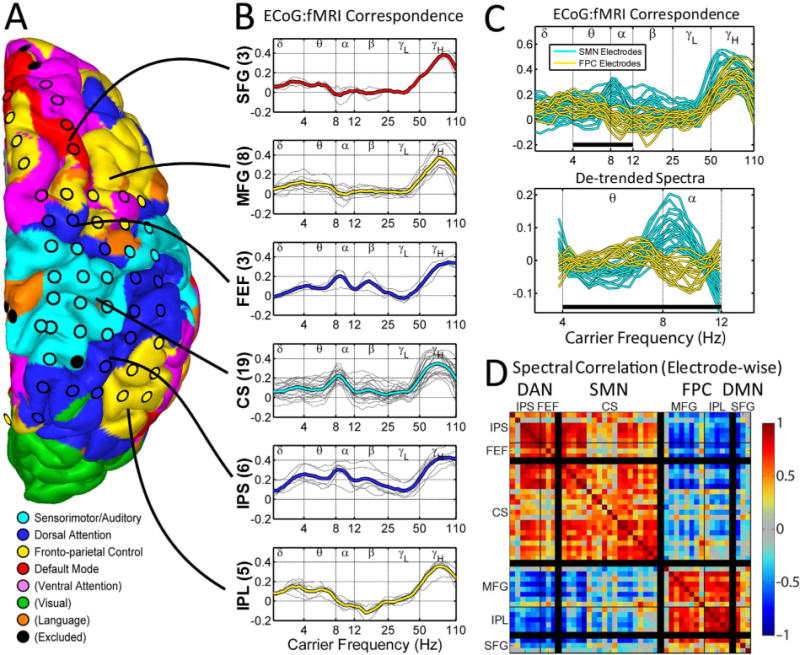
ECoG:fMRI correspondence spectra vary according to RSN. Results shown for the same subject as Fig. 2. A. RSN nodes defined within-subject by supervised classification of fMRI signal correlation patterns. B. ECoG:fMRI correspondence spectra averaged across electrodes within each node (grey traces: individual electrodes, thick line: within-node electrode average). C. Top: Correspondence spectra aggregated over SMN and FPC networks. Bottom: Correspondence spectra after linear detrending. Note a predominance FPC peaks at 6 Hz and SMN peaks at 9 Hz. Black bar indicates range of frequencies used to compute correlations in D. D. Correspondence spectrum similarity across electrode pairs computed by linear correlation over the 4–12 Hz range. SFG: superior frontal gyrus (DMN); MFG: middle frontal gyrus (FPC); FEF: frontal eye field (DAN); CS: central sulcus (SMN); IPS: intra-parietal sulcus (DAN); IPL: inferior parietal lobule (DAN).

Fig. 3 extends the analysis shown in Fig. 2 to include all electrodes in one subject. The cortical surface was parcellated into seven RSNs according to our previously reported scheme (Hacker et al., 2013). RSNs generally are comprised of spatially discontiguous regions (nodes). For example, the fronto-parietal control (FPC) RSN includes five distinct nodes within the illustrated cortical surface (color coded yellow in Fig. 3A). Thus, each electrode was assigned to one node of one RSN. Correspondence spectra were averaged across electrodes within each node. The results of this analysis (Fig. 3B) reveal the dependence of ECoG:fMRI correspondence on RSN. High correspondence in the gamma frequency range (nominally, above 45 Hz) is ubiquitous. Low correspondence is generally observed in the low-gamma frequency range (nominally, 25–50 Hz). At lower BLP frequencies, the frequency of maximal correspondence depends on RSN. Thus, components of the dorsal attention network (DAN; dark blue in Fig. 3A) exhibit high correspondence in the low-alpha range (8–10 Hz), whereas components of the fronto-parietal control (FPC; yellow in Fig. 3A) RSN exhibit maximal correspondence in the theta range (4–8 Hz).

The results in Fig. 3B suggest that the fine features (locations of peaks and troughs) of ECoG:fMRI correspondence spectra differ according to RSN in the 4–12 Hz range. To quantify this effect, we computed a numerical index of correspondence spectrum similarity for all electrode pairs. Specifically, the correspondence spectrum at each electrode was first linearly detrended within the 4–12 Hz range; the result of this process is illustrated in Fig. 3C. The Pearson correlation coefficient was then computed between detrended spectra for all electrode pairs (Fig. 3D). This matrix (Fig. 3D) demonstrates similarity of spectral features at the highest level of the RSN hierarchy: high similarity was found between all electrodes within the DAN and the sensorimotor network (SMN; extrinsic system). High similarity was also found between all electrodes within the FPC and the default mode network (DMN; intrinsic system). In contrast, low similarity was found for electrodes paired on opposing components of the hierarchy, e.g., DAN:DMN.

**Fig. 4:**
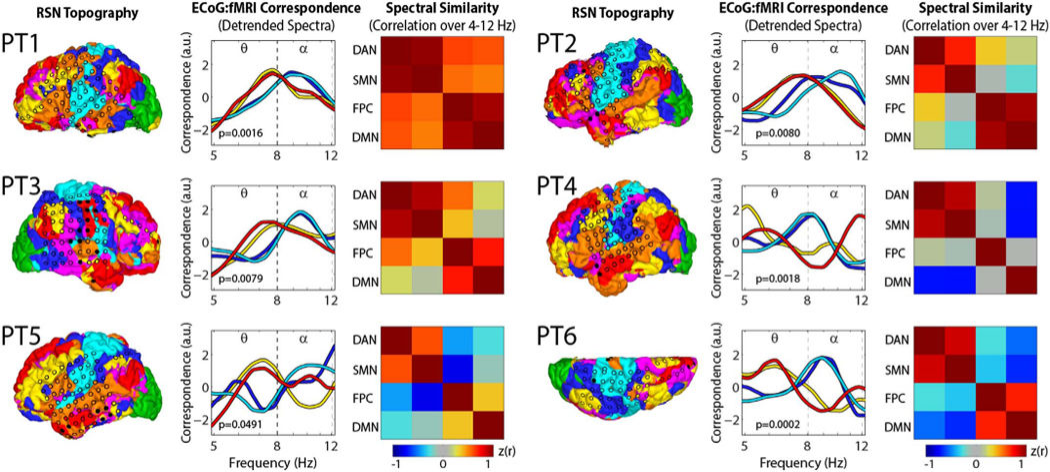
RSN coverage and spectral specificity of ECoG-fMRI correspondence for all subjects. Left Panels: Winner-take-all RSN parcellation rendered on the pial surface. Marker overlays indicate the placement of electrodes. Colors indicate the RSN to which each electrode was assigned (see Fig. 3). Middle Panels: detrended correspondence spectra averaged across all electrodes within each RSN. In this display, the detrended spectra were Z-scored. Right panels: correlation of correspondence spectra across each pair of RSN averages. P-values in middle panels indicate the statistical significance for a test of greater similarity in spectral features across network pairs that are within-system (average of DAN:SMN and FPC:DMN correlations) than across-system (average of DAN:DMN, DAN:FPC, SMN:DMN, and SMN:FPC correlations).

Similar features were observed across subjects. Fig. 4 illustrates the RSN topography and electrode coverage for each subject (left panels). Correspondence spectra for each electrode were detrended as in Fig. 3C, then averaged across all electrodes within each RSN (Fig. 4 middle panels). This analysis reveals relatively greater ECoG:fMRI correspondence in the alpha range for electrodes in the DAN and SMN and relatively greater ECoG:fMRI correspondence in the theta range for FPC and DMN. Similarity of spectral features (Fig. 4 right panels) was computed via correlation of correspondence spectra across each pair of RSN averages (analogous to correlations across electrode pairs in Fig. 3D). Within system (extrinsic, DAN:SMN; intrinsic, FPC:DMN) similarities were greater than across system (e.g., DAN:DMN) similarities in every subject. We used a permutation resampling-based approach to estimate the statistical significance of this result. Electrodes were randomly assigned RSN labels (keeping the number of electrodes per RSN constant), and the surrogate statistic for within vs. across system spectral correlation (average of DAN:SMN and FPC:DMN minus average of DAN:DMN, DAN:FPC, SMN:DMN, and SMN:FPC), was computed. 100,000 permutations were performed allowing us to estimate the null hypothesis of no significant difference. The proportion of surrogates exceeding the within vs. across similarity statistic in the real data was used to calculate subject-specific p-values (see Fig. 4), which were less than 0.05 in every subject.

The spectral specificity of resting-state BLP fluctuations across RSNs within the theta-alpha frequency range was not attributable to differences in spectral power. See Fig. S6 for power spectral density averaged across all electrodes within each RSN for each subject. It is also note that the large gamma correspondence peak, common across all networks, does not have a corresponding peak in the typical resting ECoG power spectral density.

**Fig. 5:**
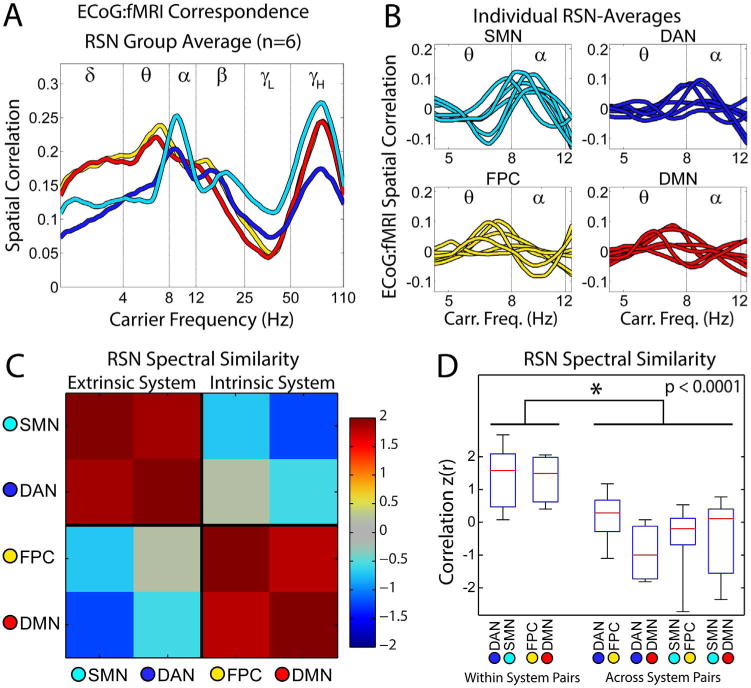
A. ECoG:fMRI correspondence spectra averaged within RSNs across all electrodes and all subjects. B. Detrended ECoG:fMRI spatial correlation spectra averaged within RSNs. Each trace corresponds to an individual participant. C. Correlation between RSN-averaged detrended spectra shown in panel (B), averaged across participants. Higher correlation (red hues) indicates greater similarity of RSN-specific spectral features. D. Distributions of inter-RSN spectral correlations over subjects. Within each box plot the red line indicates the inter-subject median, the blue box indicates inter-quartile range, and whiskers: range. Asterisk indicates significant *t*-test for greater within system vs. across system RSN spectral correlations.

Fig. 5A illustrates the group average correspondence spectrum for each RSN (across all electrodes of all subjects). Group averaged RSN-specific spectra revealed convincing system-specific features: the extrinsic system (DAN and SMN) spectra peaked at 9 Hz. The intrinsic system (DMN and FPC) spectra peaked at 7 Hz. These differences in peak loci also were consistently obtained at the single subject level (Fig. 5B). Similarity of RSN-specific spectra within and across systems was evaluated for each subject as the Fisher z-transformed Pearson correlation over log frequency spanning 4–12 Hz (Fig. 4, right panels). The RSN spectral similarity matrices were averaged over subjects to obtain the group-level results shown in Fig. 5C. These results clearly reveal a block structure corresponding to the extrinsic vs. intrinsic system dichotomy. Distributions over subjects of the RSN:RSN similarity measures are shown in Fig. 5D. At the group level, within-system correlations (average of DAN:SMN and FPC:DMN) were systematically higher than across-system correlations (average of DAN:DMN, DAN:FPC, SMN:DMN, and SMN:FPC), (p < 0.0001, one sided *t*-test). This result remained significant when estimated using the resampling approach described above (p < 10^−5^). Using rank order (Spearman’s) correlation coefficients did not significantly affect ECoG:fMRI spatial correlations. Rank order correlations were slightly weaker for high values of spatial correlation. Rank-order-based correspondence spectra had nearly identical spectral features and inter-RSN spectral correlations; greater within- vs. across-system spectral similarity remained significant (p < 0.002).

**Fig. 6:**
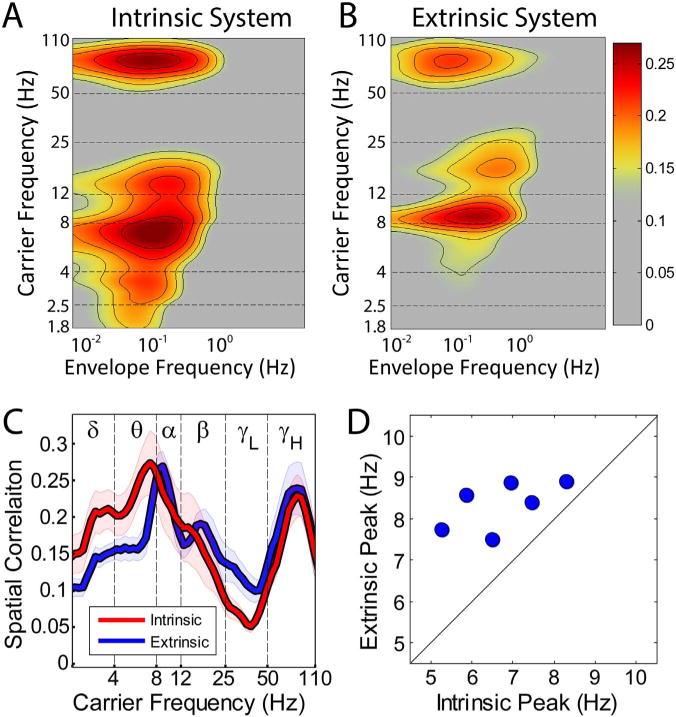
System-level spectral specificity of ECoG correlations. A. Correspondence spectrum averaged across all electrodes overlying intrinsic system RSNs (average over all subjects). Note similarity of spectral features in carrier domain to exemplar in Fig. 2C. B. Correspondence spectrum average for all extrinsic system electrodes in the same format as (A). Note similarity to Fig. 2F. A soft threshold is present in the color scale in panels A (intrinsic system) and B (extrinsic system) to emphasize the location of spectral peaks. The intrinsic and extrinsic systems exhibit a gamma spectral peak at the same frequency, but different peaks for low frequencies: 9–10 Hz for the extrinsic system average vs. 6–7 Hz for the intrinsic system average. Peak correspondence was found at similar envelope frequencies (approx. 0.1 Hz) for low and high carrier frequencies. C. Correspondence spectra collapsed over the 0.1–1 Hz modulation frequency range for both systems. Note offset of peaks in the theta and alpha range. Shaded intervals indicate standard error of the mean across subjects. D. Peak frequencies for within-subject system averages. Each marker represents the peak frequency in the 4–12 Hz range for one subject. Note systematically greater extrinsic than intrinsic peak frequency in all subjects.

Fig. 6 shows extrinsic and intrinsic system correspondence spectra averaged over all subjects. These results demonstrate, at the group level, the principle features illustrated in Fig. 2: ECoG:fMRI correspondence depends only modestly on modulation frequency but is sharply tuned in carrier frequency (panels A and B). The extrinsic and intrinsic systems both exhibit high ECoG:fMRI correspondence in the gamma (> 50 Hz) carrier frequency range and a trough at approximately 35 Hz (panel C). Most importantly, extrinsic vs. intrinsic carrier frequency specificity is most marked in the 4–12 Hz range, i.e., in the theta and alpha bands.

## Discussion

### Summary of present findings

The principal aim of this work is to determine which BLP carrier frequencies show spontaneously correlated envelope modulations in topographic correspondence with BOLD fMRI RSNs. As far as we are aware, this is the first systematic, wide-coverage examination of human ECoG:fMRI correspondence parametric in BLP carrier frequency. We find ubiquitous correspondence in the gamma band (nominally, frequencies, > 60 Hz), in accordance with prior work (He et al., 2008; Keller et al., 2013; Ko et al., 2013). Crucially, we also find correspondence in either the theta (3–8 Hz) or alpha (8–12 Hz) band, depending in whether the RSN in question falls within the intrinsic or extrinsic system. A consequence of these findings is a minimum of correspondence at in-between frequencies (centered around 40 Hz) (Fig. 6).

Prior related findings include the observation of spontaneously correlated alpha BLP as well as spontaneously correlated fast activity (gamma BLP and multi-unit firing) in homologous parts of auditory cortex (Nir et al., 2008)(Supplemental Information). These observations are consistent with the present results under the view that auditory cortex belongs to the extrinsic system. Prior related work also includes an investigation of spontaneously modulated BLP within the visual system of monkeys (Wang et al., 2012). Wang and colleagues reported alpha (8–13 Hz) much greater than gamma (30–100 Hz) BLP correlation. An emphasis on the alpha band is consistent with the present findings under the view that visual areas fall within the extrinsic system. The observation of relatively weak gamma BLP correlations is somewhat anomalous considering the available data as a whole. A possible explanation for this discrepancy is that Wang and colleagues used penetrating microelectrodes as opposed to macro-electrodes resting on the cortical surface; alpha oscillations dominate local field potentials in deeper cortical layers while gamma oscillations are more prevalent at the surface (Spaak et al., 2012) (see also *Sampling BOLD Data to Electrodes*, above).

### Cross-frequency coupling

Evidence pertaining to the spectral specificity of LFPs is contained in a large literature on phase-amplitude cross-frequency coupling (PAC). PAC refers to amplitude modulation of fast electrophysiological activity by the phase of slower activity (Canolty and Knight, 2010; Jiang et al., 2015). Theta-gamma PAC was first described as a correlate of exploratory behavior in rodents (Buzsaki, 2002; Colgin, 2013) and has since been extensively studied in humans using ECoG, e.g., (Kahana et al., 1999).^3^ It currently is widely accepted that high-frequency activity, e.g., gamma oscillations and spike discharge, reflect local processing (Buzsaki and Wang, 2012; Nir et al., 2007) whereas phase-synchronous slower activity, i.e., in the theta, alpha, beta frequency ranges (nominally, 4–30 Hz), coordinates excitability over widely separated parts of the brain (Bahramisharif et al., 2013; Burke et al., 2015; Ekstrom and Watrous, 2014; Jensen et al., 2014; van der Meij et al., 2012). This coordinating mechanism selectively links appropriate sensory, motor, task control, and memory modules according to instantaneous behavioral requirements (Fries, 2005; Helfrich and Knight, 2016). Several additional points that are crucial to the present discussion derive from the recent ECoG literature: (i) PAC is present in the resting state (i.e., in the absence of overt behavior) (Arnulfo et al., 2015). (ii) Whether the phase-defining frequency occurs in the theta or alpha band depends on brain locus. For example, in resting state ECoG data, Foster and Parvizi observed theta-gamma PAC in posterior precuneus cortex (PCC; intrinsic system), but alpha-gamma PAC in nearby visual cortex (extrinsic system) (Foster and Parvizi, 2012). (iii) The topography of correlated resting state activity matches the topography of task-induced high-frequency responses, e.g., (Foster et al., 2015; Fukushima et al., 2012).

### Relation to task-based ECoG

We interpret our results as suggesting that the intrinsic vs. extrinsic system dichotomy corresponds to the distinction between the theta-gamma vs. alpha-gamma PAC electrophysiology. If we assume that the characteristic spectral content of responses to imposed tasks also emerges spontaneously in the resting state, then support for the present perspective may be obtained by comparing our correspondence spectra to a sizeable literature on task-based (as opposed to resting state) ECoG experiments (see Roux and Uhlhaas (2014) for review). Accordingly, we review the frequency and topography of oscillatory responses induced by performance of task paradigms designed to recruit either the intrinsic or extrinsic systems.

Hippocampal theta/gamma historically has been associated with encoding and recall of memory for places and events (Buzsaki and Moser, 2013). This association was first studied in rodents but has since been amply documented in humans with implanted hippocampal microelectrodes (Kahana et al., 1999; Lega et al., 2016; Sederberg et al., 2003) as well as in humans with ECoG electrodes over retrosplenial cortex (Foster et al., 2013). Hippocampus and retrosplenial cortex unambiguously are part of the intrinsic system (Kahn et al., 2008). Theta responses induced by navigation, working memory, language, and conflict paradigms have been reported by several groups (Anderson et al., 2010; Piai et al., 2016; Raghavachari et al., 2001; Watrous et al., 2013b). The key point here is that these responses generally localize to posterior cingulate, parahippocampal, temporal, parietal, and prefrontal regions that fall within either the DMN or the FPC (compare Fig. S1 in Watrous et al. (2013b)) to Fig. 1 in Lee et al. (2012) or Fig. 4 in Doucet et al. (2011)).^4^ Theta bursting, originally observed in scalp EEG, has long been associated with conflict tasks (Cavanagh and Frank, 2014). Recent ECoG experiments definitively identify the generator of this response as the rostral cingulate zone (also known as dorsomedial prefrontal cortex), a major node of the FPC (compare Fig. 7 in Hacker et al. (2013) to Fig. 1 in Oehrn et al. (2014)). Importantly, theta burst responses induced by conflict have been recorded in human hippocampus (Oehrn et al., 2015). Moreover, midline frontal EEG theta is induced by memory as well as conflict paradigms (Hsieh and Ranganath, 2014). These observations, in aggregate, support a triple association between a particular class of cognitive operation (episodic/place memory, working memory, and conflict monitoring), theta oscillations, and the intrinsic system.

Motor behaviors suppress alpha (8–12 Hz) and beta (13–30 Hz) oscillations in association with enhanced gamma power in somatomotor cortex (Miller et al., 2007). Alpha suppression also is a correlate of auditory processing (Potes et al., 2014). Similar phenomenology as a correlate of visual processing has been frequently studied in humans using MEG and EEG (Jensen et al., 2014) but seldom using ECoG, probably because occipital electrode coverage is comparatively unusual in epilepsy surgery. It has been suggested that visual stimulation increases broadband activity in visual cortex (Winawer et al., 2013). However, Lewis and colleagues recently reported detailed observations obtained in monkeys with high-resolution ECoG grids (252 electrodes) implanted over visual cortex (Lewis et al., 2016). Analysis of the resting state ECoG data demonstrated BLP correlation topographies strikingly similar to BOLD fMRI functional connectivity within the *human* visual system (compare Fig. 2 in Lewis et al. (2016) to Fig. 2 in Buckner and Yeo (2014)). Importantly, the LFP frequencies contributing to the resting state correlations exhibited two peaks, one in the alpha/beta range, and one in the gamma range, with a clear trough in between (at ~40 Hz). Similar experimentation cannot be done in humans. However, if we assume that functional connectivity is similar in the human and the macaque (within the visual system), then the results reported by Lewis and colleagues are highly consistent with the present formulation of a correspondence spectrum, as illustrated in Fig. 6. These results further support the view that the alpha/gamma system largely corresponds to the sensory and motor areas of the cerebral cortex.

### Implications regarding selection of carrier frequency band for BLP correlation mapping

The present investigation concerns which BLP carrier frequencies exhibit envelope correlations in topographic correspondence with BOLD fMRI RSNs. We find evidence suggesting that theta and alpha BLP correlations, respectively, are present in the intrinsic and extrinsic systems. Importantly, these correlations may be sharply tuned with respect to carrier frequency, as illustrated in Fig. 2C,F and 5B. Moreover, the BLP spectral peak may vary across individuals, as shown in Fig. 6D. Thus, well-defined RSN topographies may not, in general, be obtained using BLP spanning the canonical theta (3–7 Hz) or alpha (8–12 Hz) bands. On the other hand, robust gamma-band correspondence is a ubiquitous feature of ECoG correlation maps obtained with all seeds, as shown in Fig. 6. Thus, if the primary objective of the experiment is to define the topography of resting state correlations, as in (Foster and Parvizi, 2012; Fukushima et al., 2012), then mapping with high-frequency BLP offers the most straightforward approach.

### Caveats

Observations not consistent with the present formulation can be found in the human ECoG literature. For example, (Foster et al., 2015) illustrated focal correlations between posterior cingulate cortex and angular gyrus, both nodes of the DMN, in high frequency broadband (HFB) BLP and beta BLP but not theta BLP. Although this observation does not agree with our findings, we note that it involves the PCC (where our coverage is limited) and was computed in a fixed canonical frequency band in 3 subjects. A systematic comparison to fMRI across more finely resolved frequencies and brain regions would be of interest in datasets with extensive medial coverage. Our results, in some cases, show sharply tuned spectral features narrower than canonical frequency bands (see Fig. 2 and S6). The sharpness of these features and the possibility of inter-subject frequency variability (e.g., of the alpha band, see (Haegens et al., 2014)) underscores the importance of either well resolved spectral analyses or perhaps individually tuned frequency bins.

We have suggested that task-based induced responses inform what we may expect in the resting state. In this context, we mention a task-based ECoG study incorporating impressively wide coverage obtained by pooling data from 16 subjects (Ramot et al., 2012). Induced gamma responses confirmed partition of the cortex into task positive vs. task negative regions, in accordance with our results. The low frequency induced responses within the DMN were described as occurring in the alpha/beta (not theta) band. The extent of discrepancy with the present results is unclear because, in some of the illustrated time-frequency plots, the responses actually appear to be greatest in the delta/theta range. Moreover, multiple cognitive processes were invoked, as the task required visual processing mixed with language and conflict. (Specifically, the subjects were asked to identify the class of object depicted in images that had been degraded by backwards masking.).

A related complication arises in the interpretation of oscillatory responses that change frequency, e.g., as in the experiment of (Fell et al., 2011). In this case, subjects were shown regularly paced word stimuli that had to be judged old or new. Medial temporal induced theta began about 1 s ahead of each word but accelerated into the alpha/beta band about a half second later. One interpretation of this phenomenon is that task control processes activated during word anticipation induced theta BLP after which spatial attention to the screen induced faster activity. This interpretation is entirely consistent with the conclusions reached by Voytek et al. (2010) on the basis of EEG and ECoG studies contrasting non-visual (e.g., language) vs. visual (target detection) tasks.

### Relation to MEG

Magnetoencephalography (MEG) provides access to a temporal frequency band (nominally, 1–100 Hz) inaccessible to fMRI. Hence, combining MEG with fMRI offers a potentially powerful approach to investigating brain physiology (Hall et al., 2014). The topography of task-evoked responses obtained with both modalities has been compared using relatively straightforward methods, e.g., as in Singh et al. (2002). Extending this experimental paradigm to the resting state is not straightforward because the cross-modal comparison must be made on the basis of correlation maps. Several groups have reported MEG-fMRI comparisons of resting state data using disparate computational strategies, e.g., selection of temporal epochs meeting a particular criterion of non-stationarity (de Pasquale et al., 2012), orthogonalization of inversely modeled, source space time series (Hipp et al., 2012), and normalization of the MEG data by an estimated signal-to-noise ratio (Hipp and Siegel, 2015). Arguably, the most impressive topographic match to date between MEG and fMRI RSNs has been obtained by the Nottingham group using ICA, e.g., (Brookes et al., 2011). However, the associated MEG:fMRI correspondence spectrum does not at all match Fig. 6. Specifically, this spectrum exhibits a broad unimodal peak spanning the alpha and beta ranges (nominally 10–30 Hz) and no statistically significant correspondence in either the delta/theta or gamma bands. The correspondence spectrum reported by Hipp et al. (2012) (Supplementary Fig. 3) shows separation between alpha and beta frequencies, as in current Fig. 6. However, no correspondence is reported in the gamma range. Another study designed to improve MEG:fMRI correspondence by eliminating volume conduction effects similarly reported findings largely in the alpha and beta bands and nothing in the gamma band (Marzetti et al., 2013). These discrepancies with respect to the present results most likely reflect the very different spatial specificities ECoG vs. MEG. Whereas ECoG LFPs reflect localized current sources on a millimeter scale, inverse source localization in MEG is uncertain by several centimeters (Hauk et al., 2011). Moreover, MEG is insensitive to radial current (Mosher et al., 1992), which is the likely principal source of ECoG potentials (Buzsaki et al., 2012). A full account of the differences between resting state MEG vs. ECoG as regards correspondence with fMRI remains to be elucidated.

## Conclusions

We have presented evidence suggesting that the dichotomy between the intrinsic vs. extrinsic systems corresponds to the electrophysiologic distinction between parts of the brain that communicate via theta vs. alpha phase synchrony. We have reviewed prior results consistent with this perspective as well as evidence not consistent with this perspective. Thus, our conclusions amount to the articulation of a hypothesis. Tests of this hypothesis could be obtained from additional task-based ECoG studies in humans. However, very few behavioral paradigms exclusively recruit either the intrinsic or the extrinsic system because tasks designed to engage working memory, control and language functions must interface with the subject via sensory/motor processes. These complications could be avoided by testing our hypothesis in monkeys using wide coverage, high resolution ECoG, as in Lewis et al. (2016). Ideally, the grid should provide coverage of both the intrinsic and extrinsic systems so that the topographic distribution of theta vs. alpha oscillations can be studied simultaneously in responses to task paradigms as well as in the resting state.

## Supplementary Material

1

s1

s2

## Footnotes

2 Intracranial EEG recording simultaneously with fMRI is possible but technically very challenging, hence, used only for seizure focus localization (Grouiller et al., 2011).

3 Rodent theta (6–10Hz) is faster than primate theta (1–8Hz) but the physiology and computational significance of theta-gamma PAC probably is otherwise similar (Buzsaki et al., 2013; Watrous et al., 2013a).

4 Inconsistent nomenclature persists. In the present work, we use ‘intrinsic’ vs. ‘extrinsic’. This dichotomy is essentially equivalent to ‘task-negative’ vs. ‘task-positive’ in Lee et al. (2012) or ‘S1’ vs. ‘S2’ in Doucet et al. (2011).

## Appendix A. Supporting information

Supplementary data associated with this article can be found in the online version at doi:10.1016/j.neuroimage.2017.01.054.
