# Supplementary material for: Frequency-specific electrophysiologic correlates of resting state fMRI networks
Source: Neuroimage. Author manuscript; Available in PMC 2018 Apr 1. (PMC5745814; doi:10.1016/j.neuroimage.2017.01.054)
Supplement: 1 [file NIHMS853836-supplement-1.docx]

**Supplemental Data**


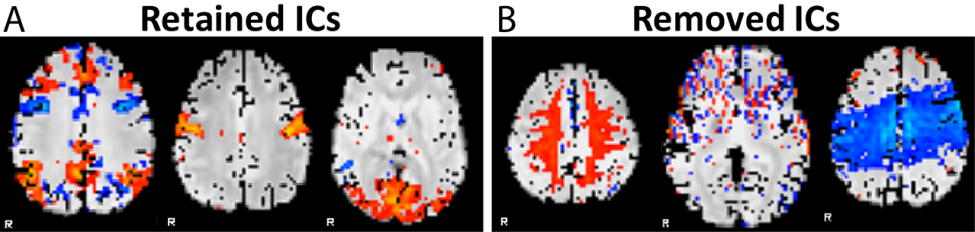


**Figure S1.** Exemplar manually classified ICA components. A. Components of neuronal origin (i.e., resting state network components). B. Artifact components of physiologic (e.g., respiratory, cardiac) or non-physiologic (e.g., head motion) origin.


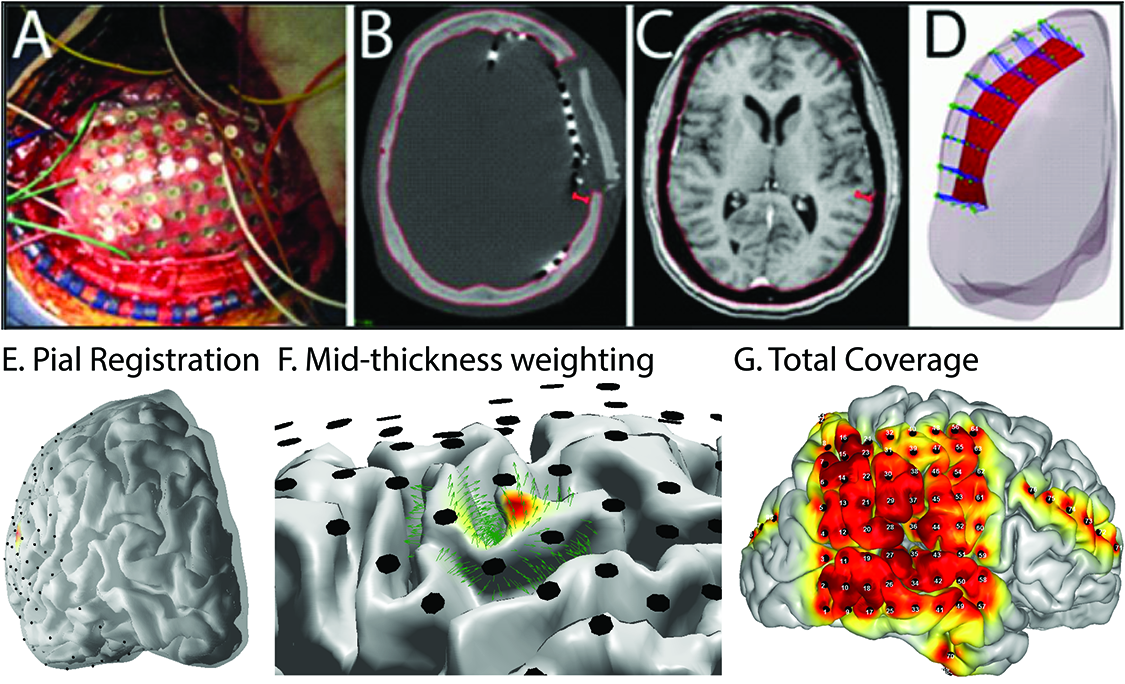
**Figure S2.** Coregistration of electrodes with brain surface. A. Intraoperative photo taken at the time electrode grid and strip implantation. B. & C. Co-registration of post-operative CT image (B) with pre-operative structural MRI (C). Electrode artifacts can be plainly seen in the CT image (B). The red outline was auto-traced on the CT and transfered to the structural MRI to verify the co-registration. Note that the electrode at the posterior skull osteotomy is deep relative to the inner table of the skull (indicated by red bar). This distortion leads to electrode coordinates recovered inside the brain in the undistorted, pre-operative MRI. D. We correct this distortion by projecting electrodes to a heavily smoothed pial surface along a vector normal to the local surface of the grid. E. Segmented electrode coordinates after projection to the smoothed pial surface (D). F. Electrode models (registered to pial surface) overlaid on mid-thickness surface. The surface sampling function for each electrode was defined as the inverse square of the distance from each electrode surface locus ($\vec{r}_{e}$) to each mid cortical thickness surface locus ($\vec{r}_{mid}$). The relative weighting function for an individual electrode (after integrating over the electrode surface, $\vec{r}_{e}$) is indicated by the surface hue. Note that the contribution to each electrode is strongly local. G. Sum of weight functions across all electrodes. This computation is not used for analyses, but is presented here to illustrate electrode coverage.


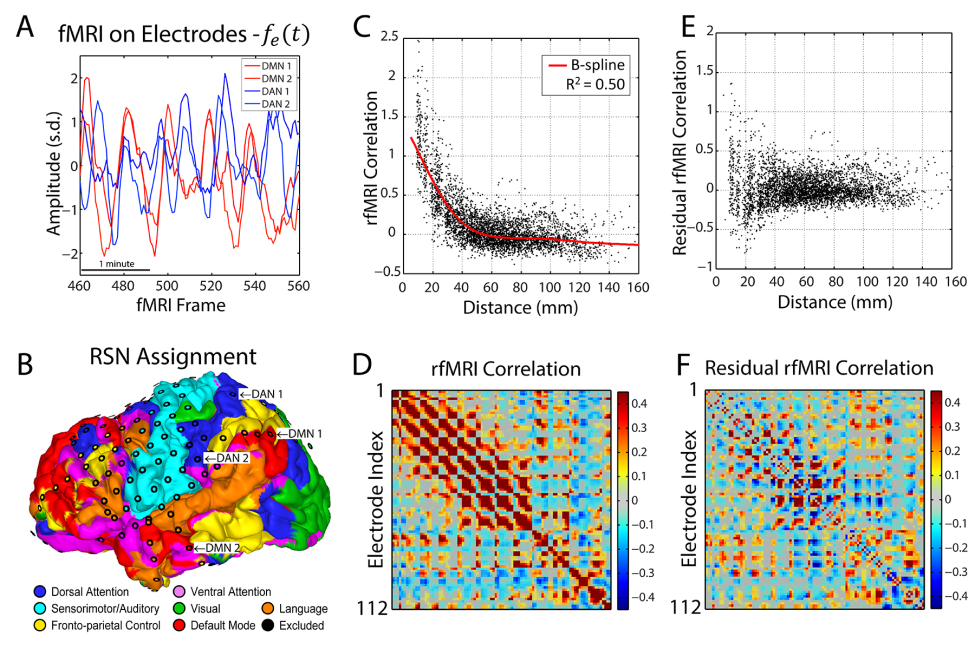


**Figure S3.** Computation of pair-wise correlations of fMRI timeseries sampled onto ECoG electrodes. A. Estimate of RSN identity of each surface locus in an individual based on supervised classification of seed-based BOLD correlation maps. B. Exemplar BOLD timeseries after sampling surface-processed timeseries to electrodes using the weighting function described in Fig. S2. C. Temporal correlation matrix of BOLD signal between all pairs of electrodes after spline regression of distance-related correlation trends (see Fig. S4C).


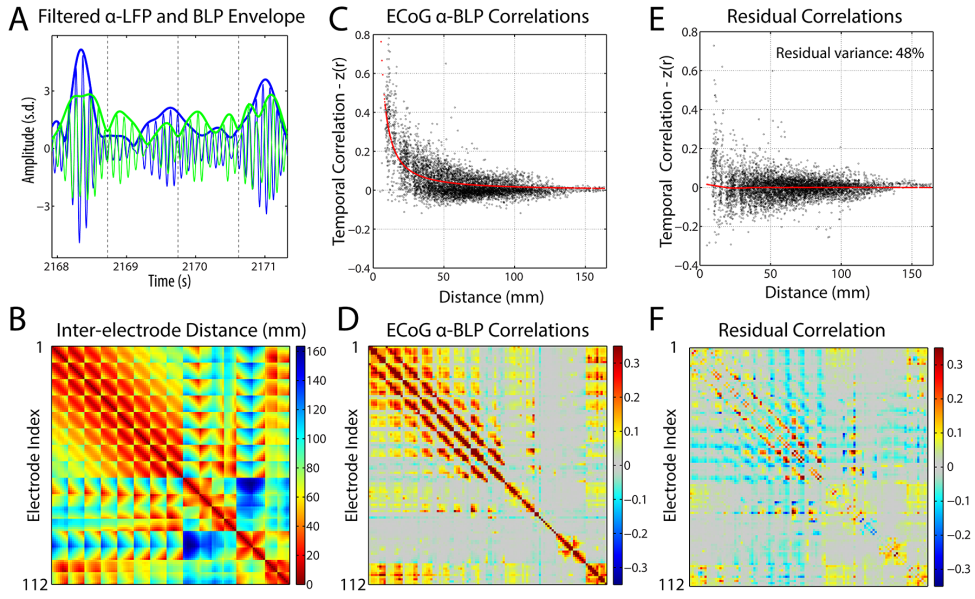


**Figure S4.** ECoG Preprocessing. A. 10 Hz signals filtered from two exemplar channels. Thin lines indicate the filtered LFP (carrier wave). Thick lines indicate the band-limited power envelope. B. Euclidean distance across all pairs of electrodes. C. Relationship of correlation values to electrode distance in raw data with a two parameter fit. D. Pairwise temporal correlation between electrodes corresponding to the data in C. E&F. Correlations after re-referencing data to a common average.


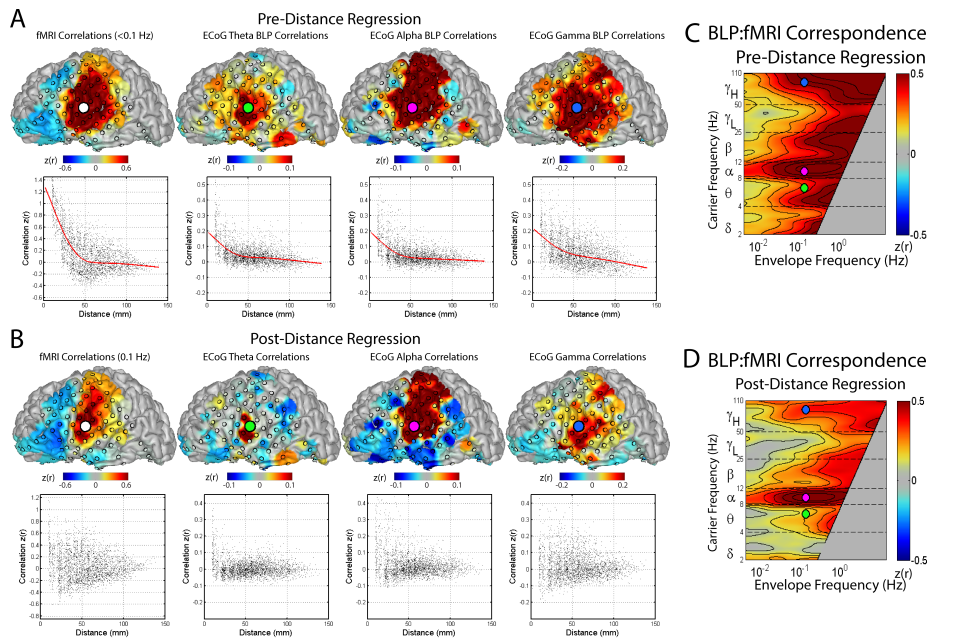


**Figure S5**. Effect of distance regression on fMRI and ECoG correlation maps. A. fMRI (<0.1 Hz) and ECoG BLP (θ, α, γ) correlations for primary motor cortex seed. Scatterplots indicate estimates of the correlation-distance relationship (for all channel pairs) and correlation-distance trend estimate (red line). B. Residual correlation maps, as per (A), after trend removal. Seed electrodes in A and B (large colored markers) indicate frequency coordinate in C and D. C. Spatial correlation of maps in (A), parametric in ECoG BLP carrier and modulation frequencies. Colored circles indicate frequencies for exemplars in (A). D. ECoG:fMRI correspondence as in (C) with distance-regression.

This figure illustrates the impact of distance regression on correlation topographies and the effect on measures of ECoG BLP:fMRI spatial correspondence. The correlation-distance trend estimates (red line in Fig. S5A) indicate that predominately local correlations are removed. This makes a significant impact on the correlation map topography for theta, removing most correlations near the seed electrode. This indicates that most of these correlations were radially symmetric, and similar in distribution to correlations found uniformly for seed electrodes throughout the brain. In alpha, the correlation:distance trend was similar; however, comparison of the alpha correlation map before and after trend removal clearly shows that features at this seed were not radially symmetric, and were not stationary (i.e., uniform throughout the brain). Accordingly, Fig. S5 C and D shows that the alpha peak in correspondence was preserved after distance regression. Thus, spectral spectificity has been enhanced after removing spectrally uniform correspondence that was the result of spatially non-specific, local correlations.


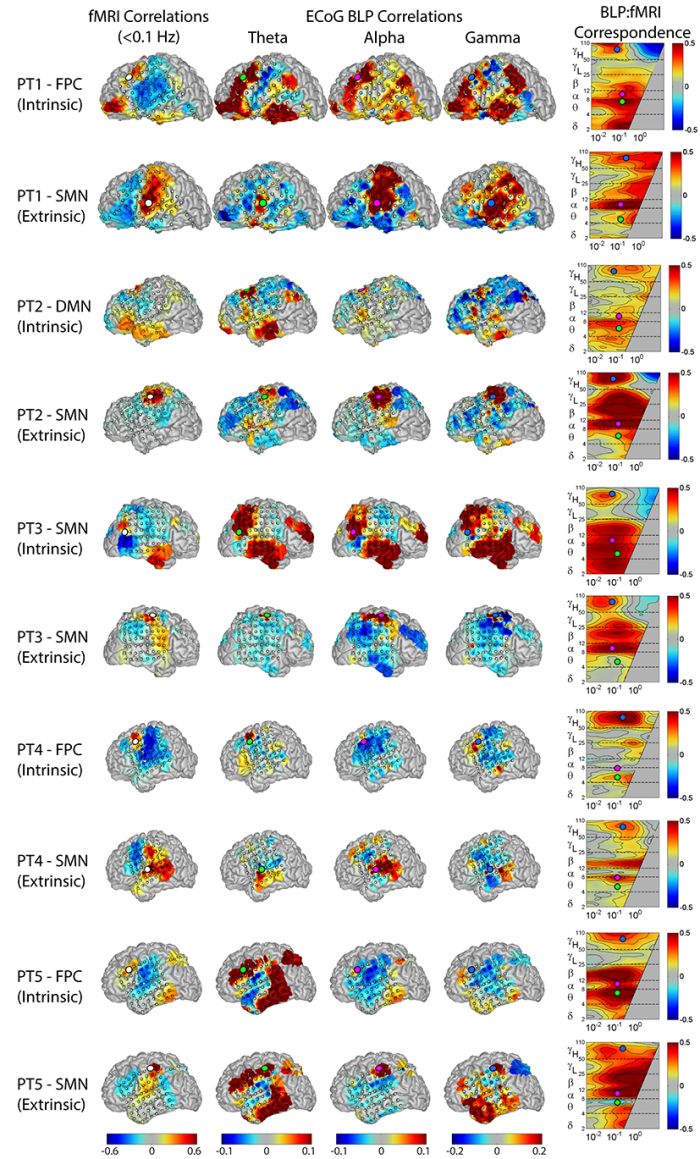


**Figure S6.** Spatial correspondence of ECoG and fMRI correlation maps in single subjects. Exemplar seeds from intrinsic and extrinsic RSN regions are illustrated for each subject. Methodology and conventions match Figure 2. Left panel: Seed-based fMRI correlation map for a seed region (white circle). Middle 3 panels: Seed-based ECoG BLP correlation maps for the same seed location as the fMRI correlation map for theta, alpha, and gamma carrier frequencies. Right panel: ECoG:fMRI correspondence assessed by spatial correlation of seed based fMRI (left panels) and ECoG (middle panels) correlation maps. Colored circles indicate ECoG frequencies used to compute correlation maps.


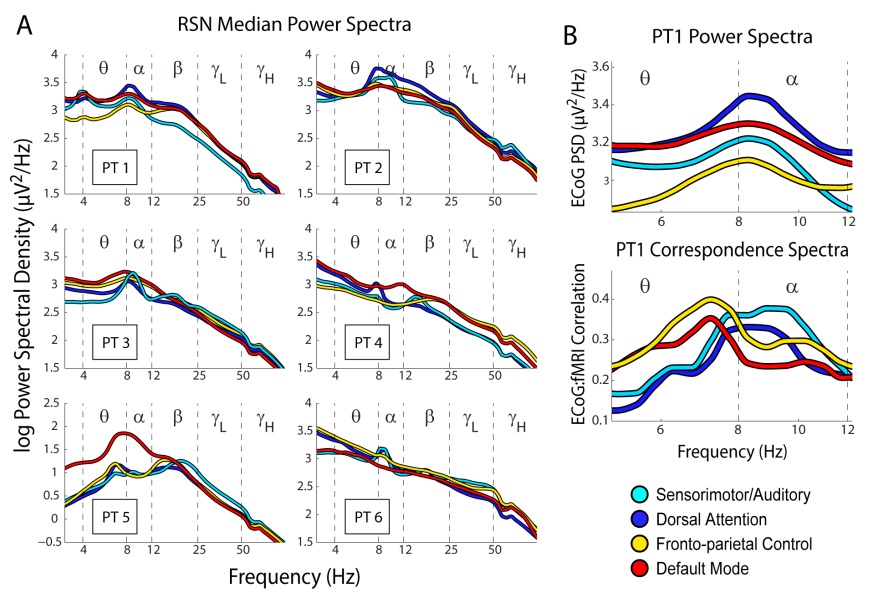


**Figure S7**. Relation of ECoG:fMRI correspondence to power spectral density (PSD) within RSNs. A. PSD was estimated in every electrode using Welch’s method (half-overlapping 1 second bins). PSD estimates were averaged across electrodes within each RSN. Oscillatory features (local PSD peaks, i.e., deviations from power law) occur in the ~5-20 Hz range. B. Comparison of PSD and ECoG:fMRI corresondence in an exemplar subject. Results in PT1 show that spectral power alone does not account for variability across RSNs of spectral peaks in ECoG:fMRI correspondence. A peak in ECoG:fMRI correspondence was reliably found in the high-gamma range (cf. Fig. 3), but no comparable feature is evident in the ECoG PSD. Some similarity was evident between ECoG PSD and the alpha peak of ECoG:fMRI correspondence spectra for the SMN and DAN. This similarity mostly likely reflects the presence of an alpha (but not theta) frequency peak in the resting state ECoG PSD.
