# Supplementary material for: Frequency-specific electrophysiologic correlates of resting state fMRI networks
Source: Neuroimage. Author manuscript; Available in PMC 2018 Apr 1. (PMC5745814; doi:10.1016/j.neuroimage.2017.01.054)
Supplement: s1 [file NIHMS853836-supplement-s1.docx]

Table S1

|  | Sex | Age | Seizure Pathology | Localization / Procedure | Outcome (Engel Class) |
| --- | --- | --- | --- | --- | --- |
| PT1 | F | 14 | Complex partial (idiopathic) | Left medial temporal lobe / lobectomy | Rare disabling seizures (II) |
| PT2 | M | 15 | Complex partial (cortical dysplasia) | Left medial frontal / topectomy | Seizure free (I) |
| PT3 | M | 19 | Complex partial (WHO I Ganglioglioma) | Right temporo-parietal junction / topectomy | Seizure free (I) |
| PT4 | M | 19 | Complex partial (WHO II oligdendroglioma) | Left inferior frontal lobe / topectomy | Rare disabling seizures (II) |
| PT5 | M | 55 | Complex partial (idiopathic) | Left temporal lobe / lobectomy | Seizure free (I) |
| PT6 | F | 12 | Simple partial (focal cortical dysplasia) | Right medial anterior parietal / topectomy | Worthwhile improvement (III) |

Table S1. Clinical subject profiles
