# Supplementary material for: Frequency-specific electrophysiologic correlates of resting state fMRI networks
Source: Neuroimage. Author manuscript; Available in PMC 2018 Apr 1. (PMC5745814; doi:10.1016/j.neuroimage.2017.01.054)
Supplement: s2 [file NIHMS853836-supplement-s2.docx]

Table S2

| Participants | | | BOLD Data | | ECoG Data | | Electrode Exclusion | | | RSN Coverage (Electrodes) | | | |
| --- | --- | --- | --- | --- | --- | --- | --- | --- | --- | --- | --- | --- | --- |
| Patient | Sex | Age | Duration (mins) | Frames Rejected | Epochs | ECoG Data (total, mins) | Total | Rejected (Imaging) | Rejected (Ephys) | DAN | SMN | FPC | DMN |
| PT1 | F | 14 | 66.0 | 5.9% | 5 | 135.6 | 100 | 24 | 0 | 10 | 15 | 15 | 13 |
| PT2 | M | 15 | 73.3 | 4.9% | 19 | 387.6 | 116 | 18 | 4 | 14 | 31 | 14 | 10 |
| PT3 | M | 19 | 58.7 | 3.6% | 15 | 703.1 | 88 | 2 | 10 | 21 | 9 | 16 | 7 |
| PT4 | M | 19 | 44.0 | 3.3% | 5 | 194.0 | 64 | 0 | 3 | 15 | 10 | 7 | 8 |
| PT5 | M | 55 | 54.9 | 5.2% | 6 | 293.0 | 84 | 7 | 2 | 9 | 11 | 10 | 8 |
| PT6 | F | 12 | 62.2 | 14.3% | 10 | 241.0 | 70 | 12 | 3 | 13 | 20 | 15 | 6 |
| Average: | | 22 | 60 | 6.2% | 10 | 326 | 87 | 11 | 3.6 | 11.2 | 12.3 | 11.8 | 8.3 |

Table S2. Patient experimental data and electrode coverage.
